# Surgical Outcomes of da Vinci Xi™ and da Vinci SP™ for Early-Stage Endometrial Cancer in Patients Undergoing Hysterectomy

**DOI:** 10.3390/jcm13102864

**Published:** 2024-05-13

**Authors:** Motoki Matsuura, Sachiko Nagao, Shoko Kurokawa, Masato Tamate, Taishi Akimoto, Tsuyoshi Saito

**Affiliations:** Department of Obstetrics and Gynecology, Sapporo Medical University, Sapporo 060-8556, Japan; nagaos@sapmed.ac.jp (S.N.); s.kurokawa@sapmed.ac.jp (S.K.); mtamate@sapmed.ac.jp (M.T.); akimotsu0622@sapmed.ac.jp (T.A.); tsaito@sapmed.ac.jp (T.S.)

**Keywords:** early-stage endometrial cancer, da Vinci Xi™, da Vinci SP™

## Abstract

**Objectives**: This study aimed to evaluate and compare the feasibility and outcomes of two robotic hysterectomy (da Vinci Xi™ vs. da Vinci SP™) systems without lymph node dissection in patients with early-stage endometrial cancer, and assess the postoperative recurrence rate and overall survival of patients. **Methods**: A retrospective review of 84 patients who underwent robotic hysterectomy for endometrial cancer (stage 1A) was conducted. Surgical procedures, patient characteristics, intraoperative measures, and postoperative outcomes were statistically analyzed. A single gynecologist performed all surgeries. **Results:** Patient characteristics, average age, and body mass index showed no significant differences between the two models. The total operative time was significantly shorter with da Vinci SP™. Recurrence was identified in only one patient operated on with da Vinci Xi™. All patients were alive during analysis, with a median overall survival of 38 and 9 months for da Vinci Xi™ and da Vinci SP™, respectively. **Conclusions:** Robotic hysterectomy without lymph node dissection appears to be a safe and effective approach for patients with early-stage endometrial cancer. The da Vinci SP offers the advantage of shorter operative times than the da Vinci Xi™. These findings support the consideration of robotic surgery as a viable option for selected patients.

## 1. Introduction

The number of endometrial cancer cases is increasing, and it is the most common cancer affecting the female reproductive organs. Endometrial cancer is the 6th most commonly occurring cancer in women and the 15th most common cancer overall [1]. There were more than 417,000 new cases of endometrial cancer in 2020 worldwide [1]. In Japan, there were 17,880 new cases of endometrial cancer and 2644 deaths in 2020 [2]. Endometrial cancer often presents with symptoms such as abnormal uterine bleeding at an early stage, resulting in a high proportion of cases being diagnosed at this stage [3].

Lymphadenectomy in endometrial cancer has diagnostic significance and influences the decision to undergo postoperative therapy because lymph node metastasis is an independent risk factor for postoperative recurrence [4]. Therefore, it is necessary to accurately determine the surgical stage. However, lymphadenectomy increases patient invasiveness, leading to an increase in the risks of lymphedema and lymphocyst formation.

For patients with grade 1 or 2 endometrioid carcinoma, estimated to be stage 1A (FIGO2008) preoperatively, lymphadenectomy may be omitted after careful evaluation. This is because the frequency of lymph node metastasis in these cases is extremely low, and the significance of lymphadenectomy as a staging procedure is reportedly low in the literature [5,6,7,8,9].

To omit pelvic lymphadenectomy, it is necessary to establish a system that can reliably preoperatively diagnose cases with a low risk of lymph node metastasis. While examining the accuracy of preoperative estimation of stage 1A cases, we reported a diagnostic accuracy rate of 90% using magnetic resonance imaging [10].

Minimally invasive surgeries for endometrial cancer include laparoscopic and robotic surgeries. In advanced countries, the proportion of robotic surgeries is increasing. In Japan, robotic surgery for low-risk endometrial cancer has been covered by national insurance since 2018, with the number of cases increasing annually [11]. Although the number of facilities omitting lymphadenectomy for low-risk endometrial cancer is increasing, policies vary among facilities, and there is an ongoing debate about whether lymphadenectomy should be performed.

The da Vinci Xi™ (Intuitive Surgical, Sunnyvale, CA, USA) received FDA clearance in April 2014 and CE marking in 2011 [12]. The da Vinci Xi™ is an advanced robotic platform designed for minimally invasive surgery. It allows for multiple robotic arms to be used simultaneously, enabling complex procedures across various surgical specialties. The da Vinci SP™ (Intuitive Surgical, Sunnyvale, CA, USA) received FDA clearance in 2018, expanding of the da Vinci system to include single-port surgery [12]. The da Vinci SP™ is a specialized version of the da Vinci surgical system that is designed for single-port surgery. It features a single robotic arm that can be inserted through a single incision, reducing the invasiveness of procedures and potentially improving patient outcomes.

At our university, we initiated robotic surgery for low-risk endometrial cancer in 2018 using the da Vinci Xi™ until April 2023, and then switched to robotic surgery using the new da Vinci SP™ from May 2023. There are few reports in the literature on the surgical outcomes using two da Vinci systems for endometrial cancer. We omitted lymphadenectomy for cases estimated to be stage 1A (FIGO2008) preoperatively and are now evaluating the postoperative recurrence rate and surgical outcomes of these cases.

## 2. Materials and Methods

### 2.1. Patients

Eighty-four patients with endometrial cancer who underwent robotic hysterectomy using the da Vinci Xi™ and SP™ between June 2018 and April 2024 at the Sapporo Medical University Hospital were retrospectively reviewed. This study targeted cases that were estimated to be 1A preoperatively and also diagnosed as 1A by postoperative pathological diagnosis. This study was approved by the Institutional Review Board of Sapporo Medical University (approval no. 352-224). All surgeries were performed by a single gynecologist specializing in gynecological cancers.

### 2.2. Surgical Procedures

Until April 2023, all surgeries were performed using da Vinci Xi™. Although da Vinci SP™ was basically selected from May 2023, da Vinci Xi™ was sometimes selected depending on the facility situation.

Total hysterectomy and bilateral salpingo-oophorectomy were performed in the patients with endometrial cancer. All endometrial cancer cases were stage 1A and no lymph node dissection was performed.

For da Vinci Xi™, five ports (5–8 mm) were inserted at the level of the navel. For da Vinci SP™, an incision of 27–30 mm was made at the umbilical base, and the port was installed.

### 2.3. Preoperative and Intraoperative Measures

Preoperative measurements included age, body mass index (BMI), gravida, parity, history of abdominal surgery, American Society of Anesthesiologists (ASA) physical status classification, and histological findings. Intraoperative measures assessed during the surgical procedures included the total operating time, intraoperative complications, estimated blood loss, intraoperative transfusion rate, and conversion to laparotomy. Regarding operating time, we compared all cases, excluded cases with a BMI of ≥40 kg/m^2^, and excluded cases with early experience of da Vinci Xi™. Intraoperative complications were defined as bowel, bladder, ureter, or vascular injuries that occurred during surgery.

### 2.4. Postoperative Measures

The postoperative recurrence rate was investigated. The overall survival and progression free survival were also examined.

### 2.5. Statistical Analyses

The Student’s t-test was conducted to evaluate the differences between the two robotic systems. Statistical significance was set at an alpha level of 0.05. All statistical analyses were performed using Statistical Product and Service Solutions software (IBM, Tokyo, Japan).

## 3. Results

Patient characteristics are shown in Table 1. Robotic hysterectomy was performed using da Vinci Xi™ in 69 patients and da Vinci SP™ in 15 patients. The average ages of the patients undergoing hysterectomy with da Vinci Xi™ and da Vinci SP™ were 58.8 and 62.8 years, respectively (*p* = 0.322), whereas their average BMI values were 27.4 and 25.9 kg/m^2^, respectively (*p* = 0.186), with no significant differences observed. Additionally, no significant differences were observed between the patients subjected to the two robotic systems in terms of gravida, parity, number of previous abdominal surgeries, or ASA physical status.

**Figure 1:**
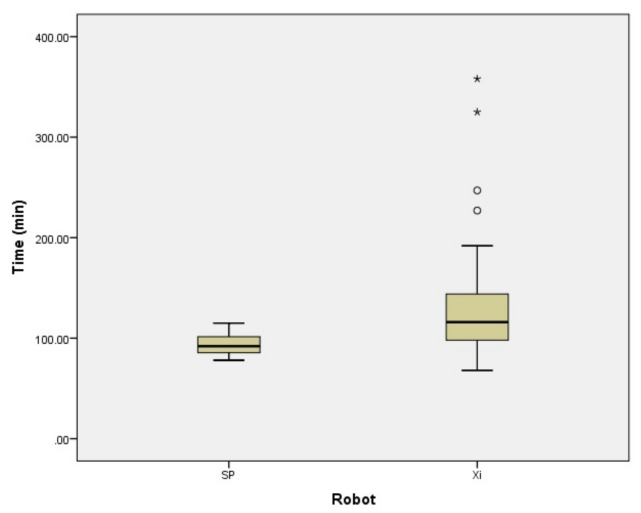
The total operative time of da Vinci Xi™ and SP™. The total operative time was 127.7 ± 51.6 and 93.7 ± 11.2 min for da Vinci Xi™ and SP™. The operation time was significantly shorter with da Vinci SP™. * and ◦ are outlier.

Table 2 shows the intraoperative surgical outcomes. The total operative time was 127.7 ± 51.6 min for da Vinci Xi™ and 93.7 ± 11.2 min for da Vinci SP™ (Figure 1). The total operative time of da Vinci SP™ was significantly shorter than that of da Vinci Xi™ (*p* = 0.013). As shown in Table 2, there was no significant difference in the weights of the removed uteri between the two groups. There was no significant difference in the amount of bleeding between the two groups. No intraoperative blood transfusion or conversion to laparotomy was observed in either model.

**Figure 2:**
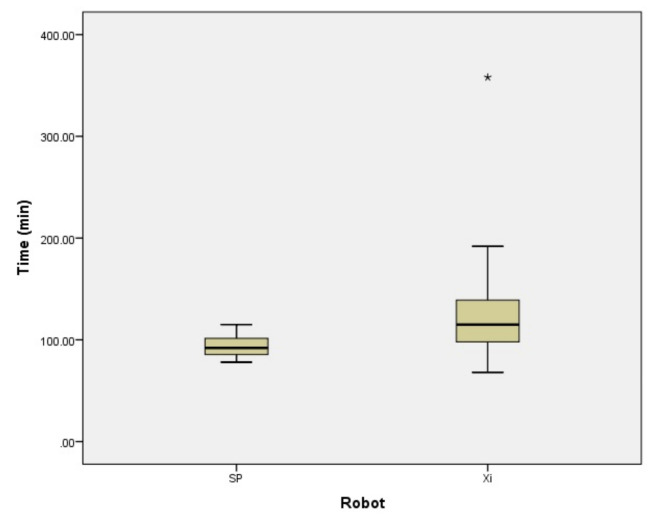
The total operative time of da Vinci Xi™ and SP™ excluding patients with BMI of ≥40 kg/m^2^. The total operative time was 120.5 ± 42.7 min for da Vinci Xi™. The operation time was significantly shorter with da Vinci SP™. * is outlier.

Of the 69 cases operated on by da Vinci Xi™, seven cases had a BMI of ≥40 kg/m^2^. Among the 15 patients who underwent surgery using the da Vinci SP™, none had a BMI of ≥40 kg/m^2^. The surgical time was compared for 62 cases, excluding these seven cases (Figure 2). The total operative time was 120.5 ± 42.7 min for da Vinci Xi™. Even after excluding obese cases, which are considered to be more difficult to operate, the operation time was significantly shorter with da Vinci SP™ (*p* = 0.023).

**Figure 3:**
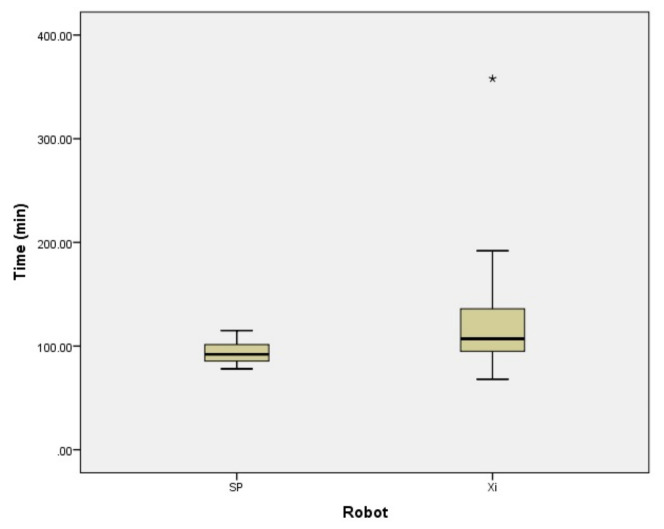
The total operative time of da Vinci Xi™ and SP™ excluding patients with BMI of ≥40 kg/m^2^ and initial 5 patients of da Vinci Xi™. The total operative time was 117.3 ± 43.0 min for da Vinci Xi™. The operation time was significantly shorter with da Vinci SP™. * is outlier.

To eliminate the influence of the learning curve, we excluded the initial 5 cases of da Vinci Xi™ and cases with a BMI of ≥40 kg/m^2^ and compared the surgical times (Figure 3). The total operative time was 117.3 ± 43.0 min for da Vinci Xi™ and 93.7 ± 11.2 min for da Vinci SP™. Even excluding initial experience, the operative time for da Vinci SP™ was significantly shorter (*p* = 0.046).

**Figure 4:**
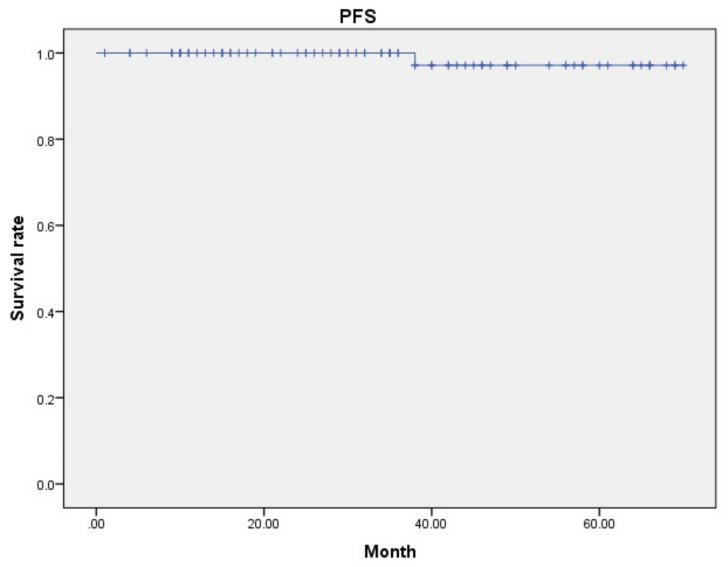
Progression free survival of all cases. Median progression free survival for all cases was 33 months.

Of the 69 patients operated on by the da Vinci Xi™, three were diagnosed with lymphovascular invasion and received postoperative chemotherapy. Of the 15 patients operated on by the da Vinci SP™, one patient was diagnosed with lymphovascular invasion and received postoperative chemotherapy. Of the 84 patients examined in this study, only one patient showed recurrence after surgery. This case was operated on by da Vinci Xi™, and the diagnosis was stage 1A with no evidence of lymphovascular invasion; however, recurrence occurred in the pelvic lymph nodes 38 months after surgery. The patient has undergone surgery and is currently alive and well without recurrence for 23 months. In the present study, the recurrence rate was 1.2%. All 84 patients examined in this study are alive, and the median OS was 38 months (range, 12–70) for da Vinci Xi™ and 9 months (range, 1–11) for da Vinci SP™. The median overall survival for all patients was 33 months (range, 1–70 months), and the median progression free survival for all patients was also 33 months (Figure 4).

## 4. Discussion

The study compared the outcomes of robotic hysterectomy using two different systems, da Vinci Xi™ and da Vinci SP™, among 84 patients with endometrial cancer of early stage. Recurrence after surgery was rare, with only one case observed, which interestingly occurred in a patient without lymphovascular invasion. However, the recurrence was managed with surgery, and the patient is currently without recurrence. This study suggests that robotic surgery without lymph node dissection is appropriate for patients estimated to have stage 1A disease before surgery.

There is extensive literature on the usefulness of lymph node dissection in advanced endometrial cancer, with many studies suggesting that lymph node dissection may detect tumor metastases early and improve patient survival [5,13]. However, the complications and side effects associated with lymph node dissection should also be considered. For example, complications from abdominal surgery and the risk of lymphedema have been reported [14]. Therefore, indications for lymph node dissection require careful assessment. It is important to consider the patient’s individual risk factors and risks associated with the procedure, and to consider the balance between benefits and risks.

The usefulness of lymph node dissection in low-risk early endometrial cancer remains controversial. Lymph node dissection is generally recommended for patients based on the stage and risk factors; however, the risk of lymph node metastasis is relatively low for early stage, low-risk endometrial cancer [5,6,7,8,9]. This finding supports the hypothesis that lymph node dissection is unnecessary. Complications and unnecessary side effects associated with lymph node dissection should also be avoided. However, some argue that lymph node dissection is important even if the possibility of lymph node metastasis is low. This is because if lymph node metastasis occurs, the treatment and prognosis may change. Additionally, there may be concerns about the risk of recurrence and worsening of prognosis at a later date if lymph node dissection is not performed.

The International Federation of Gynecology and Obstetrics (FIGO) presented recently its new 2023 staging system for endometrial cancer [15]. After 2023 FIGO staging system, the assessment of low-risk endometrial cancer includes extensiveness of lymphovascular space invasion and molecular classification. These factors are not yet included in treatment decisions. This study targets cases classified as stage 1A according to FIGO 2009 criteria. In FIGO 2023, stage 1A has been further subdivided, necessitating future consideration for determining surgical approaches.

We also conducted a comparative study between the two da Vinci models. There were no significant differences in patient age, BMI, gravida, parity, number of previous abdominal surgeries, or ASA physical status between the two groups. This suggests that the baseline characteristics of the patients were similar, which is important for comparing the outcomes between the two robotic systems. The total operative time for the da Vinci SP™ was significantly shorter than that for the da Vinci Xi™. This indicates that da Vinci SP™ may offer efficiency benefits in terms of reduced operative time, which could potentially translate to cost savings and reduced intraoperative complications. We performed surgeries with the da Vinci Xi™ when we began robotic surgery. Therefore, there might be an influence of the learning curve on the comparison of surgical times. However, even when excluding cases from the initial introduction for comparison, the da Vinci SP™ had shorter surgical times. Additionally, in considerations excluding cases of high difficulty with a BMI of 40 or higher, the da Vinci SP™ also had shorter surgical times. The short surgical time for da Vinci SP™ is thought to be due to the short time required for port insertion, roll-in, and docking.

As of the end of April 2024, da Vinci SP™ has been approved in three countries, and seven da Vinci SP™ machines have been introduced in Japan. Seon et al. reported that although da Vinci SP™ had a longer console and total operation time than da Vinci Xi™, robotic endometrial cancer surgical staging using da Vinci SP™ appears to be safe and feasible in terms of intra- and post-operative complications [16]. Miyamura et al. investigated the initial results of da Vinci SP™ in benign gynecological diseases, and reported that robotic-assisted total hysterectomy using the da Vinci SP™ allowed clinicians to safely perform surgeries according to the conventional systems [17].

Although the single-incision da Vinci SP™ is considered to be a difficult surgery, our results suggest that a surgeon who is proficient with the da Vinci Xi™ can perform the same or better surgery than the da Vinci Xi™ from the first case. These results suggest that robotic surgery using the da Vinci SP™, which omits lymph node dissection, may be effective in providing less invasive surgery for patients with low-risk endometrial cancer. The da Vinci SP™ is expected to undergo further evolution and development in the future, driven by its advanced technology and innovation. This system will serve as a foundation for enabling even more precise and efficient surgeries as medical technology continues to advance. In the future, it may incorporate higher levels of autonomy and real-time data processing capabilities, further enhancing surgical performance.

This study has some limitations. First, the sample size of da Vinci SP™ surgery was small. Second, the comparison of two model was not randomized. Third, the surgery using da Vinci Xi™ included early cases from when the surgeon started performing robotic surgery. Although it was confirmed that there were no significant differences in the backgrounds of the cases between the two groups, such points need to be taken into consideration. Forth, Lymph node biopsy is essential for accurate staging; however, in this study, lymphadenectomy and biopsy were not performed when lymphadenopathy was not detected via imaging, thus not strictly representing precise surgical staging.

## 5. Conclusions

The study suggests that both da Vinci Xi™ and da Vinci SP™ are effective and safe for robotic hysterectomy, with da Vinci SP™ offering the advantage of shorter operative times. Advancements in research and development and technological innovation are expected to allow the da Vinci SP™ to play a crucial role in gynecologic oncology. This study also suggests that robotic surgery without lymph node dissection is appropriate for patients estimated to be stage 1A before surgery. However, further research is needed to explore the potential factors influencing the long-term survival outcomes between the two systems.

## Figures and Tables

**Table 1:** Patient characteristics.

Characteristics	da Vinci Xi™ (*n* = 69)	da Vinci SP™ (*n* = 15)	*p*
Age	58.8 ± 13.9	62.9 ± 15.4	0.322
BMI	27.4 ± 8.3	25.9 ± 5.5	0.186
Gravidity	1.5 ± 1.5	1.5 ± 1.4	0.952
Parity	1.5 ± 1.1	1.4 ± 1.3	0.33
Number of previous abdominal surgeries	0.3 ± 0.5	0.3 ± 0.8	0.785
ASA physical status classification	1.5 ± 0.7	1.4 ± 0.5	0.514
ASA physical status classification, n			
1	40	9	
2	22	6	
3	7	0	

All values except ASA physical status classification are presented as mean ± SD. BMI, body mass index; ASA, American Society of Anesthesiologists.

**Table 2:** Intraoperative and postoperative surgical outcomes.

	da Vinci Xi™ (*n* = 69)	da Vinci SP™ (*n* = 15)	*p*
Total operation time, min (mean ± SD)	127.8 ± 51.6	93.7 ± 11.2	0.013
Estimated blood loss, ml (mean ± SD)	15.1 ± 54.4	5.0 ± 0	0.478
Uterus weight, g (mean ± SD)	146.5 ± 112.2	114.7 ± 50.9	0.287
Conversion to laparotomy, n	0	0	
Transfusion, n	0	0	
Total operation time, min (mean ± SD)	127.8 ± 51.6	93.7 ± 11.2	0.013
Intraoperative complication, n			
Yes	0	0	
No	69	15	
Recurrence, n			
Yes	1	0	
No	68	15	
OS, month (median, range)	38 (12–70)	9 (1–11)	

SD, standard deviation; ASA, OS, overall survival.

## Data Availability

The data used for this study, though not available in a pubic repository, will be made available to other researchers upon reasonable request. The datasets used and analyzed during the current study are available from the corresponding author on reasonable request.
